# Oviposition Traits and Vitellogenin-Related Gene Functions in *Ooencyrtus kuvanae*

**DOI:** 10.3390/insects17050468

**Published:** 2026-04-30

**Authors:** Ciding Lu, Xinyuan Zhang, Qiufang Zheng, Qunda Chen, Chuang Yan, Haoyu Lin, Zesui Chen, Feiping Zhang, Guanghong Liang

**Affiliations:** 1 Forestry College, Fujian Agriculture and Forestry University, Fuzhou 350002, China; 2210429001@fafu.edu.cn (C.L.); 12304029019@fafu.edu.cn (X.Z.); 52304022043@fafu.edu.cn (Q.Z.); 52404022036@fafu.edu.cn (Q.C.); fpzhang@fafu.edu.cn (F.Z.); 2 Forestry College, Jiangxi Environmental Engineering Vocational College, Ganzhou 341002, China; yuanchuang2008@126.com; 3 State Key Laboratory of Agricultural and Forestry Biosecurity, College of Forestry, Fujian Agriculture and Forestry University, Fuzhou 350002, China; 4 Forest Protection Research Institute, Fujian Academy of Forestry, Fuzhou 350012, China; 5 Minqing Baiyun Mountain Forest Farm of Fujian, Fuzhou 350800, China; zhengyingter6688@gmail.com

**Keywords:** *Ooencyrtus kuvanae*, vitellogenin gene, vitellogenin receptor gene, ovary, reproductive characteristics

## Abstract

*Ooencyrtus kuvanae* is an egg parasitoid species that attacks the egg masses of *Dendrolimus* spp. and *Lymantria* spp. in China, which gives it a temporal niche advantage against pest infestations by *Dendrolimus* spp. and *Lymantria* spp. Moreover, it has a short life cycle, high offspring outcome, and female-biased population, showing distinctive ovarian development and oviposition behaviors and thus providing an ideal model for reproductivity and regulatory mechanisms. Previous studies have found that the Vitellogenin (*Vg*) and Vitellogenin Receptor (*VgR*) genes play important regulatory roles in the ovarian development of a few wasp species, but little is known about how these two genes work within *O. kuvanae*. Therefore, we observed their oviposition traits, characterized their gene structure, and clarified the function of *Vg* and *VgR*. The results showed that continuous daily oviposition significantly reduced the daily mature eggs and offspring per female by providing a single host egg for oviposition in each experimental trial, while the proportion of female offspring reached 100%, indicating that thelytokous parthenogenesis occurred. The full-length sequences of *OkVg* and *OkVgR* were cloned and submitted to GenBank. qPCR detection revealed that the transcription levels were the highest in adults. Feeding 20-hydroxyecdysone can increase *OkVg* gene expression (by 2.4-fold), while feeding juvenile hormone can promote their *OkVgR* expression (by 2.3-fold). RNA interference significantly downregulated *OkVg* and *OkVgR* expression in adult ovaries. And ds*Vg* significantly reduced the ovarian egg load by 45% (*p* < 0.05), while ds*VgR* caused oviduct contraction and offspring decrease. Simultaneous silencing of *OkVg* and *OkVgR* significantly reduced offspring outcomes, indicating both genes may jointly dominate oocyte development. This study provides functional evidence of molecular regulation and interaction between *OkVg* and its receptor genes.

## Simple Summary

*Ooencyrtus kuvanae* possesses excellent traits such as productive fecundity, the ability to achieve multiple oviposition, and thelytokous parthenogenesis, making it a promising candidate for biological control of forest pests. In this work, we determined its fecundity trait and successfully cloned the sequences of Vitellogenin gene (*OkVg*, PQ565682.1) and Vitellogenin Receptor gene (*OkVgR*, PQ580724.1) from *O. kuvanae*. These two genes are mainly involved in the formation of ovarian structure and maturation of eggs within *O. kuvanae* females and synergistically dominate the regulation of oocyte development and fecundity. This study provides a new model for exploring the molecular regulatory mechanisms underlying ovarian development in egg-parasitoids and offers a critical theoretical basis and technical support for the mass rearing of *O. kuvanae* and biological control of forest caterpillars.

## 1. Introduction

*Ooencyrtus kuvanae* Howard is an important egg parasitoid that parasitizes the egg masses of several caterpillar species in forests, including *Lymantria dispar*, *Lym. xylina*, *D. punctatus*, *D. houi*, *Lycorma delicatula*, and *Halyomorpha halys*, and suppresses the pest population before infestation; thus, it has broad application prospects worldwide [1,2]. Previous studies have shown that *O. kuvanae* typically exhibits thelytokous parthenogenesis when provided with the eggs of *Lym. xylina* [3]. When using *Antheraea pernyi* eggs as hosts, each female wasp can yield 23 adult wasps from one host egg and continuously parasitize multiple eggs for 11 days [4]. Even under changing conditions, it can lay eggs and is normally able to complete its life cycle, indicating that it has an extremely strong oviposition capacity and adaptability. However, little is known about the development of *O. kuvanae* eggs and their regulatory modes.

Generally, reproductive strategies are critical for the survival and thriving of parasitoid populations, which are closely related to their biocontrol efficacy in practice. Most parasitoid species engage in sexual reproduction, whereas only a few species can facultatively perform parthenogenesis under specific conditions [5,6]. This parthenogenesis can significantly enhance population fitness under limited resources (e.g., male scarcity and patchy host distribution) and environmental stress (e.g., fluctuations in temperature and humidity) [7,8]. Basically, the fecundity of parasitoid is mainly reflected in the number of eggs and offspring quantity. Each female can produce mature eggs to expand the offspring population if enough host eggs are provided. Some previous studies have indicated that Vitellogenin (*Vg*) and Vitellogenin receptors (*VgR*) undergo metabolism and synthesis under the action of various compounds and hormones (e.g., ecdysteroids (20E) and juvenile hormone (JH)) within the ovary, and a single mature egg is fully developed [9,10]. *Vg* is a material prerequisite for egg synthesis and provides essential substances, such as nutrients, for embryonic development [11,12]. *VgR* mediates *Vg* endocytosis and transports it into oocytes. *VgR* plays a key role in regulating egg development, and its expression reaches the highest level when the egg approaches maturity [13,14].

To date, various parasitoid species globally have played an important role in biological control [15,16]; however, only nine genes of *Vg* and 13 genes of *VgR* from 30 species, such as *Pteromalus puparum*, *Nasonia vitripennis*, *Encarsia formosa*, *Copidosoma floridanu*, and *Trichogramma dendrolimi* [14], have been identified, respectively, based on the NCBI gene database. Moreover, there is species specificity in the transport mechanisms and expression patterns of these genes among different species [17,18].

Therefore, in this study, we observed the ovarian development and reproductive characteristics of *O. kuvanae*, and further revealed the *Vg* and *VgR* that regulate reproductive traits. The sequence characteristics and expression patterns of these genes were analyzed, and finally, their effects on ovarian development and reproductive capacity were verified. This result will theoretically be helpful for revealing the reproductive strategies and egg synthesis mechanisms of *O. kuvanae*.

## 2. Materials and Methods

### 2.1. Insect Rearing

Host egg masses of *Lym. xylina* were collected from Pingtan Island (119°46′42″ E, 25°36′48″ N), Fujian, China, and stored in a constant-temperature incubator (25 °C, RH 80%, photoperiod 14L:10D) until parasitoids emerged. Mass rearing of *O. kuvanae* was performed using *A. pernyi* eggs as hosts. Newly emerged adult wasps were fed 10% honey water (10 mL water + 1 mL pure honey). Parasitoids and *A. pernyi* eggs (used as hosts) at a ratio of 1:2 (wasp:egg) were placed together for two days of parasitization. Then, *A. pernyi* eggs were removed and incubated under a constant-temperature incubator until the next generation of adult wasps emerged.

### 2.2. Oviposition Characteristics

Newly emerged *O. kuvanae* adults were reared individually in 5 mL Eppendorf tubes (25 °C, 80% humidity, light cycle 14L:10D). Fresh honey water and one host egg were supplied daily until the adult died. The number of offspring that emerged from the parasitized eggs was counted daily. Additionally, the dead females were individually dissected to count the eggs left in the ovaries.

Considering that vitellogenin, under the regulatory effect of hormones, affects the egg maturation and oviposition capacity of adult wasps, exogenous hormones were added to the nectar-water diet of newly emerged adult wasps (honey water = HW, juvenile hormone = JH, 20-hydroxyecdysone = 20E): 1%JH (0.01 g of JH + 1 mL of 10% HW) and 1% 20E (0.01 g of 20E + 1 mL of 10% HW). All female wasps were divided into two groups. One group was observed to determine their egg load and the other was immediately exposed to *A. pernyi* eggs at a ratio of 1:1. Finally, the exposed eggs were reared in an incubator until adult offspring emerged.

### 2.3. Acquisition of the Vitellogenin and Vitellogenin Receptor Sequences

Total RNA from the ovaries of *O. kuvanae* (100 individuals in total) was extracted using an Eastep^®^ Super Total RNA Extraction Kit (cat. no. LS1040; Promega Corporation, Madison, WI, USA). RNA degradation and purity were determined using 1% agarose gel electrophoresis and a NanoDrop spectrophotometer (Thermo Fisher Scientific, Waltham, MA, USA). First-strand cDNA was synthesized using a Tiangen^®^ FastKing RT Kit with gDNase (Tiangen Biotech Co., Ltd., Beijing, China), following the manufacturer’s instructions. The synthesized cDNA was stored at −20 °C for subsequent experiments.

Specific primers (Table S1) were designed with reference to the *Vg* and *VgR* gene sequences of other parasitoid wasp species retrieved from the National Center for Biotechnology Information (NCBI) database (https://www.ncbi.nlm.nih.gov/; retrieval date: 20 June 2024). PCR amplification was performed using an Applied Biosystems 2720 Thermal Cycler (Thermo Fisher Scientific, Singapore) to obtain conserved fragments of the *Vg* and *VgR* genes from *O. kuvanae*.

According to the instructions of the Vazyme^®^ HiScript-TS 5′/3′ RACE Kit, 3′ RACE-ready cDNA was synthesized, followed by the synthesis of 5′ RACE-ready cDNA. RACE-specific primers were designed based on previously obtained conserved gene fragments (Table S2). The synthesized gene fragments were amplified and purified using the Universal DNA Purification Kit, then connected with a 5 minTMM TA/Blank Zero Cloning Kit (Vazyme Biotech Co., Ltd., Nanjing, China), and finally transformed into chemically competent Escherichia coli DH5α cells (Sangon Biotech Co., Ltd., Shanghai, China). Transformants (manifested as white colonies) were screened on LB agar plates containing ampicillin (Amp^+^), and positive clones were selected by PCR using vector universal primers (M13-F and M13-R). Bacterial strains containing positive clones were submitted to Sangon Biotech (Shanghai) Co., Ltd. for Sanger sequencing.

The obtained sequencing reads were assembled using DNAMAN 9 software, and the assembled sequences were aligned with the original conserved fragments using the “Multiple Sequence Alignment by Florence Corpet” tool (http://multalin.toulouse.inra.fr/multalin/, accessed on 6 March 2024) to confirm and obtain full-length *Vg* and *VgR* sequences. BLAST (https://blast.ncbi.nlm.nih.gov/Blast.cgi, accessed on 6 March 2024) alignment was performed using the NCBI database. The complete sequences were subjected to comparative analysis with sequences already deposited in GenBank via BLASTP search. Ultimately, the full-length *Vg* and *VgR* gene sequences of *O. kuvanae* were submitted to GenBank, and accession numbers were obtained.

### 2.4. Bioinformatics Analysis of Ovarian Development Genes in O. kuvanae

The molecular weights (kDa) and isoelectric points (pI) of *Vg* and *VgR* proteins were predicted using the ProtParam program (ExPASy, https://web.expasy.org/protparam/, accessed on 12 March 2024). The N-terminal signal peptides of *Vg* and *VgR* were predicted using SignalP 4.1 (https://www.cbs.dtu.dk/services/SignalP/, accessed on 12 March 2024). Multiple amino acid sequences from species with high similarity were obtained using the NCBI online BLAST alignment. The Multalin software (http://multalin.toulouse.inra.fr/multalin/, accessed on 12 March 2024) was used to realign these amino acid sequences to achieve consistent results. The domains of the *Vg* and *VgR* sequences were predicted using SMART Domains (https://smart.embl.de/, accessed on 12 March 2024), and the domains of highly similar species were compared. Finally, a species phylogenetic tree was constructed using the Maximum Likelihood method in MEGA X, incorporating *Vg* and *VgR* genes from *O. kuvanae* and other insects (Tables S3 and S4). Bootstrap values obtained from 10,000 replicates were labeled at the nodes of the phylogenetic tree.

### 2.5. Temporal Expression Dynamics of Vg and VgR and Their Sensitivity to Hormones 

The temporal expression of *Vg* and *VgR* genes was determined to identify the precise period for interference via injection. *O. kuvanae* samples were collected at different developmental stages, including early instar larvae (4 d after parasitism), late instar larvae (6 d after parasitism), young pupae (8 d after parasitism), old pupae (14 d after parasitism), and newly emerged adults (0 d after emergence). Emerging wasps were fed 10% honey water (HW), 1% ecdysone (20E), and 1% juvenile hormone (JH), and adults after continuous parasitism for 2 d (Ad3) to determine the sensitivity of adults to key hormones. The collected samples were stored in liquid nitrogen and transferred to a −80 °C freezer.

Real-time fluorescent quantitative primers were designed with Primer Premier 5 based on the obtained *Vg* and *VgR* sequences, and Glyceraldehyde-3-Phosphate Dehydrogenase (GPD) of *O. kuvanae* was used as a reference gene (Table 1).

For quantitative real-time PCR, the reaction system was prepared as follows: 12.5 µL of SYBR Premix Ex TaqII (Takara Bio Inc., Kusatsu, Shiga, Japan), 2 µL of cDNA template, 0.4 µL of forward primer, 0.4 µL of reverse primer, and 9.47 µL of ddH_2_O. The reaction conditions and annealing temperature were set based on the melting temperatures of the primers. The reaction program was as follows: pre-denaturation at 95 °C for 10 min, followed by 40 cycles of denaturation at 95 °C for 10 s, annealing at the specific temperature of the quantitative primers for 10 s, and extension at 72 °C for 20 s. A melting curve was generated by holding the temperature at 95 °C for 10 s and increasing the temperature from 65 °C to 95 °C at a rate of 0.55 °C per step, with each step held for 5 s. The relative expression levels were calculated using the 2^−ΔΔCt^ method [19], and the obtained data were subjected to significance difference analysis.

### 2.6. Functional Verification of Vitellogenin and Vitellogenin Receptor Genes

#### 2.6.1. Synthesis and Immersion of *Vg*-dsRNA, *VgR*-dsRNA, and *GFP*-dsRNA

Using the ORF sequences of *Vg*-dsRNA and *VgR*-dsRNA as templates, specific primers were used to amplify the PCR products of the two genes (the primer sequences are shown in Table 2). The products were separated and recovered using 1.5% agarose gel electrophoresis. ds*Vg*, ds*VgR*, and *GFP* double-stranded RNA (ds*GFP*) were synthesized using the T7 RiboMAX^TM^ Express RNAi System (Promega Corporation, Madison, WI, USA). The quality was tested and the concentration was recorded. The synthesized dsRNAs were stored at −80 °C for later use.

Parasitized *A. pernyi* eggs were selected for rearing and dissected after 13 d of parasitism to obtain wasp pupae (compound eyes brown) under a stereomicroscope (Carl Zeiss AG, Oberkochen, Germany) [20]. Thirty pupae were collected and transferred onto a solid medium composed of 2% agar and 0.5 g streptomycin, with three replicates for each treatment. Subsequently, 10 µL of ds*Vg* or ds*VgR* was slowly injected into each pupa. An equal volume of ds*GFP* was used as the negative control, and an equal volume of distilled water was used as the positive control.

#### 2.6.2. Silencing Effect of RNAi on Ovarian Development in *O. kuvanae*

*O. kuvanae* pupae injected with *Vg*-dsRNA and *VgR*-dsRNA emerged as adults at 48h after injection. Total RNA was extracted from the ovaries of *O. kuvanae* adults and reverse-transcribed into cDNA at 12, 24, and 48 h after emergence. The expression levels of *Vg* and *VgR* were detected using qPCR, with the *O. kuvanae* GFP gene as the reference gene and the *GFP*-dsRNA group as the control.

#### 2.6.3. Parasitic Capacity Monitoring of *O. kuvanae*

The same batch of *O. kuvanae* pupae injected with *Vg*-dsRNA and *VgR*-dsRNA, as described in Section 2.6.1, were reared until adults emerged, and the ovaries of females were dissected to observe egg maturity, total eggs, mature eggs, and the proportion of mature eggs.

*O. kuvanae* adults that emerged from pupae injected with *Vg*-dsRNA and *VgR*-dsRNA were individually placed into 2 mL centrifuge tubes with 10% honey water. Each wasp was supplied with one *A. pernyi* egg, and then the parasitized eggs were replaced every 24 h. The parasitized eggs were placed in an artificial incubator at 25 ± 1 °C, with 80 ± 5% relative humidity and a 14L:10D photoperiod, to obtain total offspring until the wasps died naturally.

### 2.7. Data Statistics

All statistical values are expressed as mean ± standard error (SEM). Tukey’s honestly significant difference (HSD) *t*-test was used to analyze the effects of temperature on *O. kuvanae* reproduction, the effects of hormone feeding on reproduction, and the expression profiles of *Vg* and *VgR*. Student’s *t*-test was applied to detect differences in the expression levels between the *GFP* gene and the other two genes after dsRNA injection, with significance levels denoted as follows: * *p* < 0.05, *** *p* < 0.001, and ns for no significant difference. Statistical analyses were performed using SPSS Statistics 20, and line graphs and bar charts were plotted using Origin 2021 software.

## 3. Results

### 3.1. Reproductive Characteristics of O. kuvanae

**Figure 1:**
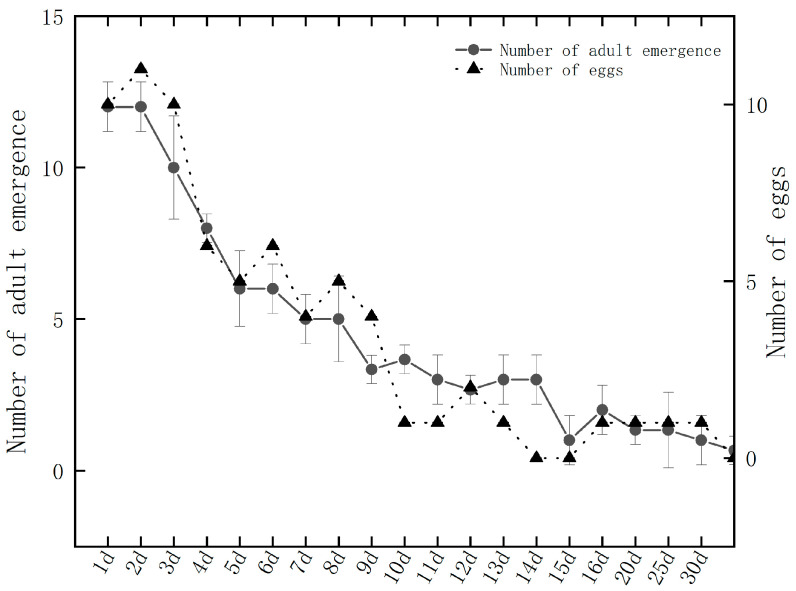
Daily number of offspring produced by *O. kuvanae*. “Number of eggs” represents the number of eggs laid per wasp per day.

When host eggs were individually provided every day, each female wasp exhibited oviposition behavior multiple times daily. A single *O. kuvanae* female could produce a maximum of 103 offspring during its entire lifetime, with 100% of the offspring being female (Figure 1). Specifically, the number of emerged offspring was the highest (more than 10 individuals) in the second-day samples. With continuous daily parasitization, the eggs in the ovaries were gradually depleted until only 1–2 offspring emerged from the host egg after 30 days (Table S5).

During the early emergence stage (Day 1) and under the condition of feeding with pure water, the average number of mature eggs (fecundity) per female was 3.40, but no oviposition occurred. Feeding on honey water and two hormones (20E and JH) significantly increased the number of mature eggs (maximum mature eggs, 17) and offspring (average offspring, 11.76) (Table 3).

**Figure 2:**
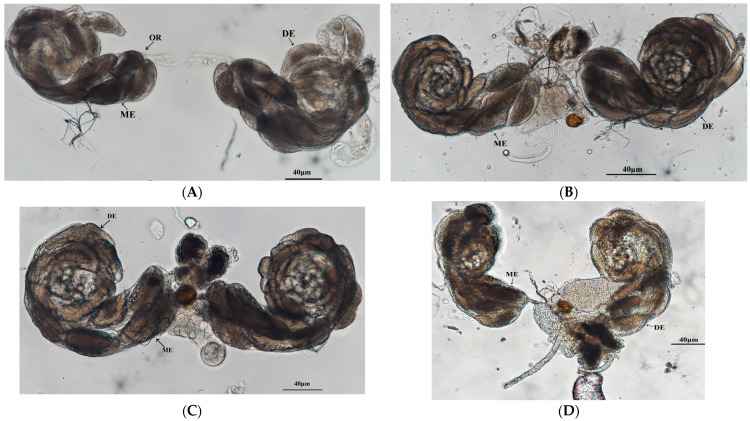

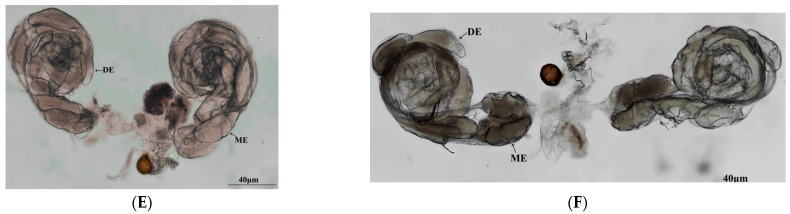
Daily ovarian development dynamics of O. kuvanae after oviposition. (**A**) Before parasitism and feeding for 2 d. (**B**) Parasitism for 1 d. (**C**) Continuous parasitism for 2 d. (**D**) Continuous parasitism for 3 d. (**E**) Continuous parasitism for 5 d. (**F**) Continuous parasitism for 7 d. Ovarioles = OR; developing eggs = DE; mature eggs = ME.

Structurally, *O. kuvanae* possesses four ovarioles in total. During the early emergence stage (Day 1), each ovariole contained one mature egg on average (approximately 41.7 μm in diameter) without oviposition (Table 3, Figure 2A). After nutrient supplementation, the mature eggs (nine) and total eggs (23) in the ovaries gradually increased during the second or third day, while the mature egg size increased (75 μm), but the number of ovarioles remained the same (Figure 2B). The number of mature (22) and total eggs (44) in the ovaries peaked at amounted 48 h post-emergence, while the mature eggs reached an optimal size (diameter of 75.6 μm) (Figure 2C). However, as the frequency of oviposition increased, the mature eggs in the ovaries gradually decreased (Figure 2D–F). During the 2 days before oviposition, the average number of mature eggs in the female’s ovaries could reach 12, while only 1–2 eggs remained within the ovary by Day 10 before oviposition ceased.

### 3.2. Sequence Analysis of the Vitellogenin Gene in O. kuvanae

One *OkVg* gene was obtained and submitted to the NCBI database (accession number PQ565682.1). The full-length sequence was 5527 bp, with an ORF of 5433 nucleotides encoding 1810 amino acids. The predicted molecular weight of the *OkVg* amino acid sequence was 204.55 kDa, and the isoelectric point was 8.36 (slightly alkaline).

**Figure 3:**
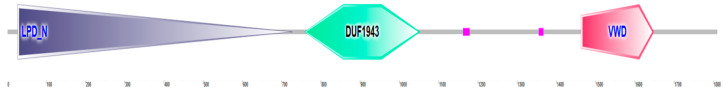
Protein domains of the *OkVg* derived from *O. kuvanae*. *OkVg* Lipoprotein N-terminal Domain (LPD_N) (700 AA), DUF1943 domain (293 AA), and von Willebrand factor (vWF) type D domain (VWD) (185 AA).

The *OkVg* domain of *O. kuvanae* showed structural similarity to several parasitoid species, including *Nasonia vitripennis*, *Ceratosolen solmsi*, and *Pteromalus puparum*, consisting of three key regulatory elements: the Lipoprotein N-terminal Domain (LPD_N), DUF1943 (involved in egg yolk protein formation from *Vg* precursors), and the von Willebrand factor (vWF) type D domain (VWD) (Figure 3). Among these domains, the VWD domain is probably involved in the specific recognition and binding of vitellogenin to oocyte receptors, which is crucial for inducing embryonic development and forming mature egg grains.

**Figure 4:**
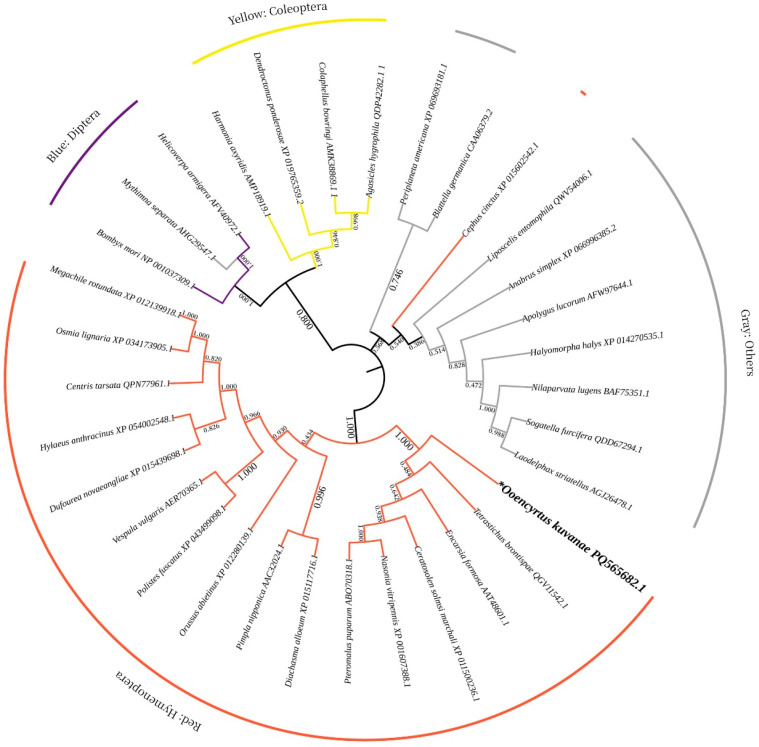
Phylogenetic analysis of *OkVg* derived from *O. kuvanae* (designated as *). A molecular phylogenetic tree was constructed based on the vitellogenin protein sequences. orange: hymenoptera; purple: diptera; yellow: coleoptera; gray: others.

Compared with other insect species, *OkVg* within Hymenoptera species were grouped into one clade, indicating an obvious similarity in their amino acid sequences (Figure 4). In terms of branch distance, *O. kuvanae* was closest to *Tetrastichus brontispae*, with a sequence identity of 100%, indicating a close phylogenetic relationship between them. Furthermore, *O. kuvanae* was relatively closely related to some parasitoid species, especially *T. brontispae*, which had the highest genetic similarity, probably indicating similar *Vg* occurrence mechanisms.

### 3.3. Sequence Analysis of the Vitellogenin Receptor Gene in O. kuvanae

One *OkVgR* gene was obtained and submitted to the NCBI database (accession number PQ580724.1). The full-length sequence was 5964 bp, with an ORF of 4173 nucleotides encoding 1390 amino acids. The predicted molecular weight of the *OkVgR* amino acid sequence was 154.23 kDa, and the isoelectric point was 5.04 (slightly acidic).

**Figure 5:**
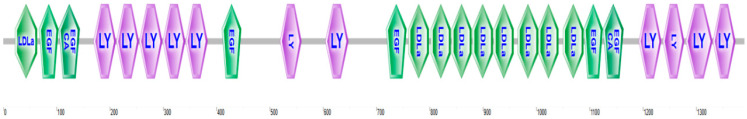
Protein domains of the *OkVgR* derived from *O. kuvanae*. *OkVgR* contains low-density lipoprotein receptor domain class A (LDLa) (41 AA), epidermal growth factor-like domain (EGF) (36 AA), calcium-binding EGF-like domain (EGF_CA) (39 AA), and low-density lipoprotein receptor YWTD domain (LY) (41 AA).

The *OkVgR* domain of *O. kuvanae* showed structural similarities to those of *C. floridanum*, *Anastatus japonicus*, and *N. vitripennis*. *OkVgR* comprises four core functional modules: low-density lipoprotein receptor domain class A (LDLa), epidermal growth factor-like domain (EGF), Calcium-binding EGF-like domain (EGF_CA), and low-density lipoprotein receptor YWTD domain (LY) (Figure 5). Among these, the 11 LY domains within the ORF account for the largest proportion in the full-length *OkVgR* sequence. As the key module responsible for specific ligand recognition, LY domains can specifically bind to *OkVg* in the hemolymph, playing a critical role in supplying nutrients for oocyte development, as well as in the parasitism and fecundity of *O. kuvanae*.

**Figure 6:**
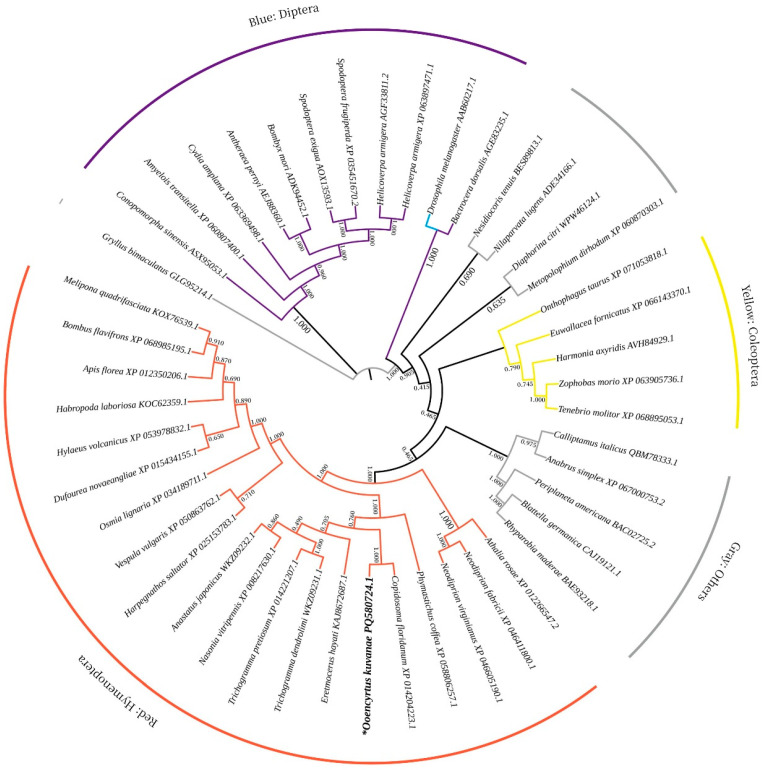
Phylogenetic analysis of *OkVgR* derived from *O. kuvanae* (designated as *). A molecular phylogenetic tree was constructed based on the protein sequences of the vitellogenin receptors. orange: hymenoptera; purple: diptera; yellow: coleoptera; gray: others.

Compared to other insects, the *OkVgR* of parasitoid species clustered into one clade, indicating similarities in their amino acid sequences (Figure 6). In terms of branch distance, *O. kuvanae* was closest to *C. floridanum*, with the highest sequence identity of 100%, suggesting that *O. kuvanae* and *C. floridanum* have the highest genetic similarity and similar mechanisms for the occurrence of *Vg* genes. However, there is currently no evidence to support the functional identification of VgRs in *C. floridanum*.

### 3.4. Temporal Expression Dynamics of OkVg and OkVgR and Their Sensitivity to Hormonal Regulation

**Figure 7:**
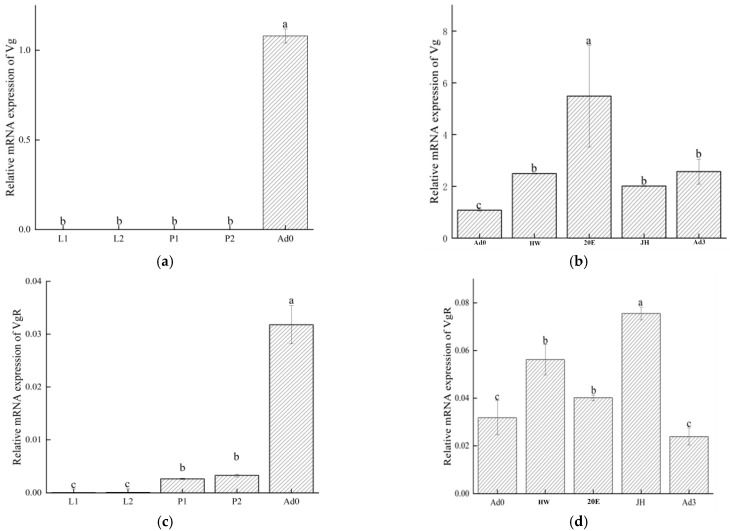
Developmental expression profile of *OkVg* and *OkVgR*, and in different feeding states of females. (**a**) To assess the development of *OkVg* expression, female larvae ranging from day 4 to 6 post-parasitization (dpp) (designated as L1–L2), pupae ranging from 8 to 14 dpp (designated as P1–P2), and newly emerged females (Ad0) were collected. (**b**) For feeding states on *OkVg* expression, the female is fed 10% honey water (designated as HW), 1% molting hormone (designated as 20E), and 1% juvenile hormone (designated as JH) after parasitism (designated as Ad3). (**c**) Developmental *OkVgR* expression. (**d**) Feeding state on *OkVgR* expression.

The expression levels of both *OkVg* and *OkVgR* in *O. kuvanae* adults were significantly higher than those in other developmental stages (*p* < 0.05). *OkVg* expression was extremely low during the larval and early pupal stages, with no significant difference (*p* > 0.05). The expression patterns of *OkVgR* and *OkVg* showed similar trends (Figure 7a,c). It is inferred that the synthesis of embryonated eggs within *O. kuvanae* may initiate from the pupal stage, while massive expression of *OkVg* and *OkVgR* occurs in the early stage of adulthood.

*OkVg* expression levels increased significantly when adult wasps were fed honey water or honey water supplemented with hormones (*p* < 0.05). The *Vg* content reached the highest level after 2 days of feeding on honey water plus 20E, and the *Vg* content decreased significantly (*p* < 0.05) after 3 days of oviposition. Similarly, the expression level of *VgR* increased significantly (*p* < 0.05) when adult wasps were fed honey water plus JH, and the expression level of VgR gradually decreased as oviposition ended (3 days post-parasitization) (Figure 7b,d).

Therefore, *O. kuvanae* primarily acquires nutrients (via feeding on honey water) during the adult stage, which is essential for egg maturation and preparing conditions for oviposition and parasitism. Furthermore, 20E and JH supplements specifically promoted the expression of *OkVg* and *OkVgR*, respectively. Finally, the expression levels of both genes decreased after oviposition, and the number of eggs synthesized within the ovaries also decreased accordingly. This finding further confirms that *OkVg* and *OkVgR* promote the synthesis of mature oocytes.

Data (mean ± SEM) represent three biological replicates with three technical replicates. Different letters above the bars indicate significant differences in *OkVg*/*OkVgR* levels in different tissues of the ovary (Tukey’s honestly significant difference test, analysis of variance, *p* < 0.05).

### 3.5. Silencing Effect of RNAi on OkVg and OkVgR in O. kuvanae

**Figure 8:**
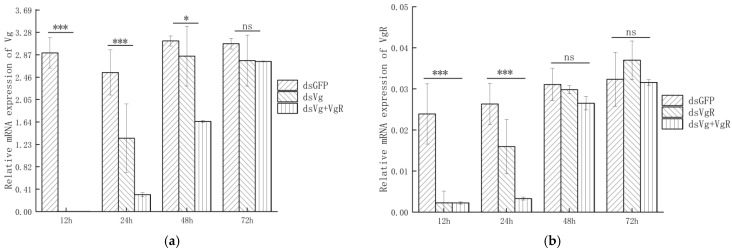
Relative expression (mean ± SEM) of *OkVg* (**a**) and *OkVgR* (**b**) at 12–72 h after dsRNA microinjection. * *p* < 0.05; *** *p* < 0.001; ns—no significant difference compared to ds*GFP* (Student’s *t*-test).

Compared with the ds*GFP* control group, *Vg* expression decreased remarkably after dsVg was injected into the old pupae of *O. kuvanae* for 24 h (*p* < 0.05, with a significant reduction of 47.26–99%, Figure 8a). Subsequently, *Vg* expression gradually increased and showed no significant difference from the ds*GFP* control group (*p* > 0.05) between 24 and 72 h post-injection (Table S6).

Similarly, the expression trend of *OkVgR* injection was similar to that of *OkVg*, which could effectively reduce the expression level of the target gene (*OkVgR*) within 24 h, with a significant reduction of 39.44–90.58% (Figure 8b, *p* < 0.05). Moreover, the expression levels of both *OkVg* and *OkVgR* decreased significantly (*p* < 0.05) when these two dsRNAs (*OkVg* and *OkVgR*) were co-injected into the *O. kuvanae* pupae.

These results indicate that dsRNA injection can effectively downregulate the expression levels of *OkVg* and *OkVgR* within 24 h, thereby inhibiting the phenotypic development of ovarian tissues in *O. kuvanae*.

### 3.6. RNAI-Induced Phenotypic Changes in O. kuvanae

**Figure 9:**
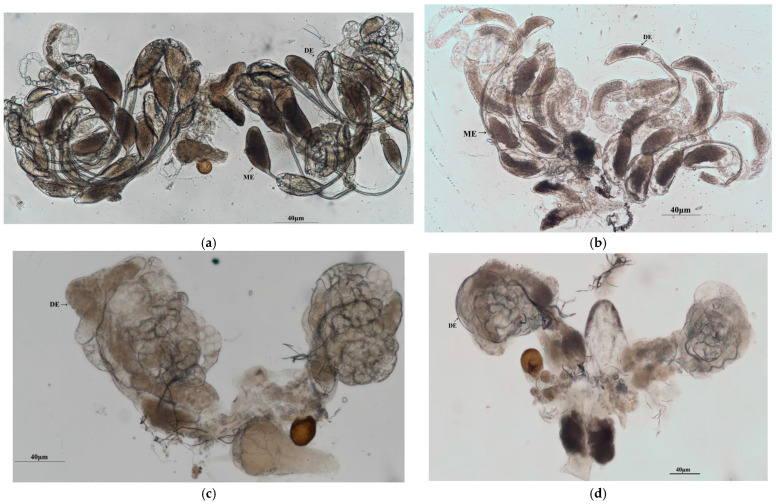
Ovary phenotypes. Ovaries were dissected from newly emerged female adults post-ds*Vg*/*VgR* or both injections and visualized under a Nikon stereo microscope (Tokyo, Japan). (**a**) Ovarian morphology of adult wasps injected with ds*GFP*; (**b**) ovarian morphology of adult wasps injected with ds*Vg*; (**c**) ovarian morphology of adult wasps injected with ds*VgR*; (**d**) ovarian morphology of adult wasps injected with both ds*Vg* and ds*VgR*. developing eggs = DE; mature eggs = ME.

Compared with the ds*GFP*-injected control group (Figure 9a), the ovarian structure and offspring number of emerged adult wasps exhibited significant changes when these two dsRNAs (*OkVg* and *OkVgR*) were injected into *O. kuvanae* pupae.

Based on ovary structure, ds*Vg* injection resulted in loose and shrunken ovarioles within adults but did not impair egg maturation or the number of mature eggs (16), which was comparable to the control group (Figure 9b). Notably, the number of mature eggs was significantly reduced (with only 1–2 mature eggs produced), and the oviducts were shrunken (Figure 9c) in adults injected with ds*VgR*.

The ovarian structural changes were similar to those observed in the ds*VgR*-injected group (Figure 9d) when *OkVg* and *OkVgR* were silenced simultaneously via co-injection of their dsRNAs.

**Figure 10:**
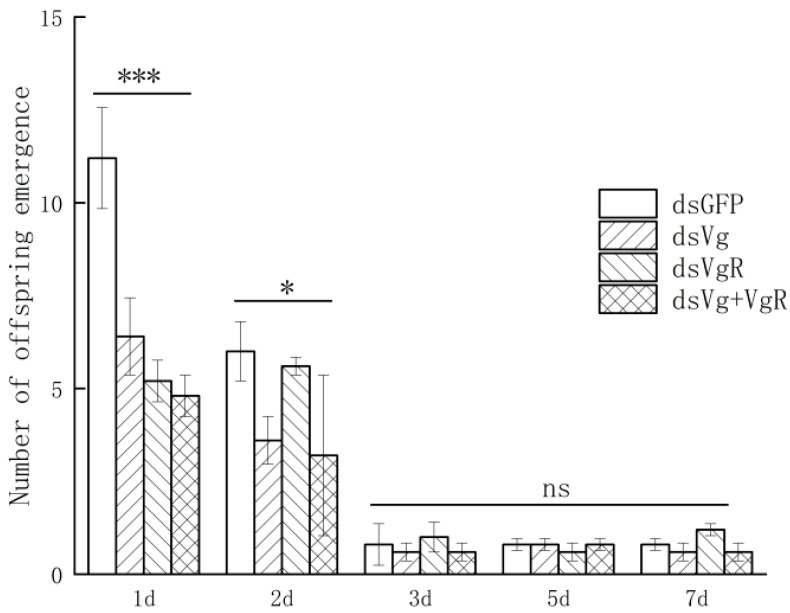
The number of mature eggs per ovary in newly emerged females following injection with ds*Vg*, ds*VgR*, or a combination of both ds*Vg* and ds*VgR*. * *p* < 0.05; *** *p* < 0.001; ns—no significant difference compared to ds*GFP* (Student’s *t*-test).

In terms of offspring individuals, silencing *OkVg* only, silencing *OkVgR* only, and co-silencing both genes all caused a significant reduction in the number of offspring produced by female adults, with a 51.19% reduction compared to the control group (*p* < 0.05, Figure 10) on the first day. On the second day post-emergence, silenced *OkVg* wasps still produced significantly fewer offspring than the control group, with an average reduction of 43.33% (*p* < 0.05). However, there was no significant difference in the number of offspring between the *OkVgR*-silenced wasps and the control group (*p* > 0.05). Subsequently, there was no significant difference in the number of offspring among all treatment groups (*p* > 0.05) after three days, probably due to the degradation of the injection effect.

## 4. Discussion

Parthenogenesis is widespread among most insect species. In parasitoid species, the induction of thelytokous parthenogenesis occurs after meiosis of germ cells: under the induction of automixis and symbiotic bacteria, gametes undergo self-replication, enabling their offspring to maintain the original diploid state [19,21]. Traditional evolutionary theory suggests that parthenogenesis causes more genetic defects and harmful mutations than sexual reproduction, resulting in potential disadvantages. However, recent studies have revealed that parthenogenetic insect populations exhibit high stability and disease resistance [22,23], which is advantageous to their survival. An increasing number of parasitic wasps have been found to use thelytokous parthenogenesis as a reproductive strategy. It has been revealed that by reducing the frequency of courtship and mating with males, these wasps can allocate more energy to host searching, oviposition, and reproductive development. This significantly lowers the production costs of mass rearing, enabling the implementation of biological control techniques more sustainably and efficiently in forest environments.

*O. kuvanae* is an important egg parasitoid attacking lepidopteran and hemipteran species. It is characterized by thelytokous parthenogenesis [1,24]. It has a temporal advantage in biological control because it targets pests before they cause damage. Previous studies have primarily focused on the biocontrol potential and host recognition mechanisms of *O. kuvanae*, while its reproductive mechanism remains unclear. *O. kuvanae* is extremely small, and the final instar pupae and ovarian tissues are only 0.3 mm in length. This poses significant challenges for the collection of samples required for gene interference injection, and means that said gene interference has a relatively low success rate. Despite the gradual application of advanced gene-editing technologies in insects, RNAi technology remains the most commonly employed tool in contemporary research focusing on parasitoid wasps. However, physical damage due to factors such as needle size, injection angle, position, flow rate, and concentration during injection is a prominent technical issue [25].

For ovarian development genes, we found that *O. kuvanae* has a structurally complete *Vg* gene whose domains are highly similar to those of other parasitoid species. However, there were differences in nutrient supply mechanisms during embryonic development [26]. Feeding two common insect hormones showed that the expression level of the *Vg* gene in *O. kuvanae* significantly increased after feeding ecdysone, and feeding juvenile hormone had no effect on the *Vg* expression level, indicating that ecdysone promotes *Vg* synthesis in *O. kuvanae*. Consistent with previous studies in other parasitoid wasps, juvenile hormone significantly promoted Vg expression; notably, *VgR* expression was also significantly upregulated (Figure 7d), suggesting that JH coordinately activates both *Vg* synthesis and *VgR*-mediated oocyte uptake to support ovarian development, as reported in *Dipetalogaster maxima* [27].

Knocking down the *Dpp*, *Punt*, *Mad*, and *Medea* genes of the ecdysteroid biosynthesis signaling pathway reduced the expression level of *Vg* within *Colaphellus bowringi*, which in turn inhibited the development of ovarian tissue [28]. In Apolygus lucorum, silencing AlPLCγ (a key signaling molecule involved in 20E signal transduction, rather than a transcription factor) downregulated the expression of AlTre-1 and AlVg, causing multiple reproductive and physiological disorders, including reduced fecundity, shortened adult lifespan, decreased egg-hatching rate, and delayed oocyte maturation [29]. This study also verified this phenomenon; when *O. kuvanae* was fed on ecdysteroids, *Vg* synthesis was promoted, consequently increasing both *Vg* expression levels and the number of offspring. Similar findings have been reported in *P. puparum*, *Bombus terrestris*, and *Apis mellifera*; *Vg* expression levels were not enhanced in individuals fed juvenile hormone. Notably, *Vg* synthesis within *P. puparum* was inhibited by feeding on high concentrations of juvenile hormones [30,31].

**Figure 11:**
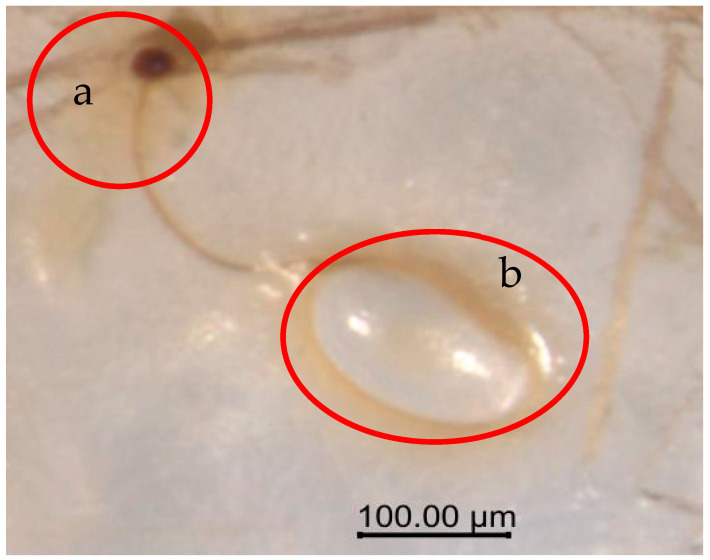
Morphological structure of *O. kuvanae* eggs. Note: (**a**) air tube; (**b**) *O. kuvanae* egg.

Recent studies have shown that although *Vg* gene loss is common in endoparasitic parasitoids, *O. kuvanae*, an endoparasitic parasitoid, retains a complete *Vg*/*VgR* system [32]. This contradiction may be related to its unique reproductive strategy: *O. kuvanae* achieves rapid reproduction through parthenogenesis, depending on the enclosed nutrient environment inside host eggs, and the eggs it lays are clearly observable (Figure 11). In contrast, the larval parasitoid wasp *Habrobracon hebetor* directly deposits embryos (without forming discrete egg structures) into host larvae, and its larvae can directly absorb the host’s hemolymph to support their own development [33]. Notably, *Vg* in *Laodelphax striatellus* not only provides nutrients for embryonic development but also carries the endosymbiont *Wolbachia* into offspring eggs during *Vg* transport, thereby completing vertical transmission [34]. When *O. kuvanae* collected from Fujian Province was mass-reared indoors, it exhibited thelytokous parthenogenesis. This trait may be closely associated with the role of symbiotic microorganisms, such as *Wolbachia*, and *Vg* plays a transport and transmission role in this process, providing an important clue to explain thelytokous parthenogenesis in *O. kuvanae*.

Similar to other parasitoid species, *O. kuvanae* possesses a conserved *VgR*-specific protein. Compared to the *Vg* gene, *VgR* exhibits a higher degree of conservation, leading to extensive research on its function. In this study, the adult stage was the main period in which *VgR* synthesis occurred, with a small amount synthesized in the last instar pupae. However, there was no significant expression of the *Vg* gene during this pupal stage, suggesting the existence of other synthetic pathways in this species. *VgR* in female adult *Trichogramma dendrolimi* shows significantly high expression and promotes the initial oviposition amount, which is consistent with the findings of this study. Additionally, this study revealed that the co-interference of *Vg* and *VgR* had a significant impact on the number of offspring produced in the first and second oviposition events in *O. kuvanae*, rather than only affecting the first oviposition. *LbVgR* does not affect ovarian development in *Leptopilina boulardi*; injecting dsRNA does not significantly decrease egg maturation but affects host-searching and courtship behaviors [35]. In contrast, mature *O. kuvanae* do not require courtship or mating and parasitize hosts once their eggs are mature. High expression of *OkVgR*, a marker of mature egg development, plays an important role in this process. Feeding on both hormones promoted *OkVgR* synthesis, but feeding on juvenile hormones alone significantly enhanced *OkVgR* synthesis to a greater extent, suggesting a new regulatory mechanism for improving the reproductive potential of wasps. When the *O. kuvanae* population degenerates or its fecundity is low, appropriate feeding of trace amounts of juvenile hormones promotes *OkVgR* synthesis, thereby enhancing fecundity.

This study is the first attempt at a combined interference determination of *Vg*/*VgR*, and the phenotypic changes were consistent with those of ds*VgR* interference. Currently, there are no reports on the simultaneous interference with these two genes. In addition, based on the conservation of *Vg* and *VgR*, we speculate that they play a role in immune function. During the growth and development of parasitoids, *Vg* provides trace elements, aids in the development of blood cells, and is indirectly involved in the immune process [36]. *Vg* and *VgR*, as key genes in vitellogenesis and ovarian development, are worthy of further in-depth research with regard to their relationships with hormone regulation, adult nutrition, and mating behavior. Further studies are needed to provide a theoretical basis for improving the reproductive potential of wasps.

## Figures and Tables

**Table 1:** Information on the qPCR primers.

Primer Name	Primer Sequences
qGPD-3′	TCACGGCTCAGGATGGTATGC
qGPD-5′	GGAAACGACCTTGTGGCTTGG
qVg-3′	CAGTCAGAGGTATATGTGGCG
qVg-5′	GAGCTGGTCCTTTACACGTAG
qVgR-3′	GCATCCACATCTACCACTCG
qVgR-5′	GCTCCTTATCCAACGTACAGG

**Table 2:** Information on dsRNA primers.

Primer Name	Primer Sequences
ds*Vg*-T7-F	GGATCCTAATACGACTCACTATAGGTATGGATGCTTTATTTGA
ds*Vg*-T7-R	GGATCCTAATACGACTCACTATAGGATACTTGCTGGTGGTTTA
ds*VgR*-T7-F	GGATCCTAATACGACTCACTATAGGAAAATCCAGTTGCGTGC
ds*VgR*-T7-R	GGATCCTAATACGACTCACTATAGGGGTTTCTCTGTGTCTTCCGT
ds*GFP*-T7-F	GATCACTAATACGACTCACTATAGGGAGACACAAGTTCAGCGTGTCCG
ds*GFP*-T7-R	GATCACTAATACGACTCACTATAGGGAGAGTTCACCTTGATGCCGTTC

**Table 3:** Effects of hormone feeding on the reproduction of *O. kuvanae*.

Feeding Hormone	Eggs	Development Time (d)	Emerging Adults
No honey or only water	3.40 ± 0.03b	/	0b
10% HW	16.71 ± 0.49a	16.29 ± 0.20	11.29 ± 0.49a
1% JH + 10% HW	17.00 ± 0.44a	16.67 ± 0.44	11.67 ± 1.11a
1% 20E + 10% HW	17.00 ± 0.60a	16.30 ± 0.21	12.33 ± 0.44a

Different letters above the table indicate significant differences (*p* > 0.05). Honey water = HW; juvenile hormone = JH, 20-hydroxyecdysone = 20E.

## Data Availability

The original contributions presented in the study are included in the article, further inquiries can be directed to the corresponding author(s).

## Supplementary Materials

The following supporting information can be downloaded at: https://www.mdpi.com/article/10.3390/insects17050468/s1, Table S1. Specific primers used in the cDNA amplification; Table S2. Primers used in the Open-Reading Frame amplification; Table S3. Species used to construct a Vg phylogenetic tree; Table S4. Species used to construct a VgR phylogenetic tree; Table S5. The detailed egg-laying data; Table S6. the microinjection efficiency and survival rates.
